# Rivermouth Alteration of Agricultural Impacts on Consumer Tissue δ^15^N

**DOI:** 10.1371/journal.pone.0069313

**Published:** 2013-07-31

**Authors:** James H. Larson, William B. Richardson, Jon M. Vallazza, John C. Nelson

**Affiliations:** Upper Midwest Environmental Sciences Center, United States Geological Survey, La Crosse, Wisconsin, United States of America; University of Otago, New Zealand

## Abstract

Terrestrial agricultural activities strongly influence riverine nitrogen (N) dynamics, which is reflected in the δ^15^N of riverine consumer tissues. However, processes within aquatic ecosystems also influence consumer tissue δ^15^N. As aquatic processes become more important terrestrial inputs may become a weaker predictor of consumer tissue δ^15^N. In a previous study, this terrestrial-consumer tissue δ^15^N connection was very strong at river sites, but was disrupted by processes occurring in rivermouths (the ‘rivermouth effect’). This suggested that watershed indicators of N loading might be accurate in riverine settings, but could be inaccurate when considering N loading to the nearshore of large lakes and oceans. In this study, the rivermouth effect was examined on twenty-five sites spread across the Laurentian Great Lakes. Relationships between agriculture and consumer tissue δ^15^N occurred in both upstream rivers and at the outlets where rivermouths connect to the nearshore zone, but agriculture explained less variation and had a weaker effect at the outlet. These results suggest that rivermouths may sometimes be significant sources or sinks of N, which would cause N loading estimates to the nearshore zone that are typically made at discharge gages further upstream to be inaccurate. Identifying definitively the controls over the rivermouth effect on N loading (and other nutrients) will require integration of biogeochemical and hydrologic models.

## Introduction

Terrestrial land cover is often strongly related to the supply of essential elements (nutrients) in nearby aquatic ecosystems (e.g., [1]–[3]). One consistently observed relationship is between agriculture and the nitrogen (N) isotopic composition of dissolved N, seston and consumers (e.g., [4]–[6]). This relationship is strong because agricultural N sources have a distinct isotopic ratio relative to other N sources [4]. However, aquatic processes can remove large quantities of N (e.g., denitrification) and in locations where these processes are prominent the movement of agricultural N downstream may be reduced and/or its isotopic signature altered [7]. Previous studies have suggested that depositional habitats (where waters slow) can significantly alter N dynamics [7].

Among aquatic ecosystem types, streams have the shortest water retention time, and thus it is unsurprising that watershed agriculture and the N isotopic composition of stream consumers is strongly correlated (e.g., [4], [8]). Other ecosystems are less cleanly connected to their upstream watersheds [7], [8]. The connection between terrestrial agriculture and nutrient loading into the nearshore zone of large lakes and oceans is particularly interesting, as these coastal areas are economically important. However, estimates of nutrient loading to nearshore areas are often made in association with monitoring stations located upstream of any direct lake or ocean influence [9]. These estimates do not include the effect of low-flow, depositional areas associated with the rivermouth itself, where water residence times are longer than in streams[10], [11]. These wetlands and embayments associated with rivermouths and estuaries may significantly alter nutrient delivery to the nearshore [12], [13]. The gap in monitoring between the river and the nearshore corresponds to a gap in the understanding of nutrient delivery to nearshore zones.

In a recent manuscript [8], we reported a relationship between agriculture and N isotopic composition of consumers in tributary rivers of Lake Michigan. That study suggested the influence of agriculture was disrupted by significant N sources or processing occurring within the rivermouth [8]. In other words, loading estimates of N to the nearshore of Lake Michigan might be inaccurate if the effect of rivermouth processing is not included. The conclusions of that study were tentative for several reasons (e.g., small sample size and limited spatial extent), but the implications if accurate are significant. For example, if certain rivermouths provide a degree of buffering to nearshore zones from upstream agricultural inputs, then short-term and long-term nutrient reduction strategies may opt to incorporate this information into prioritization schemes. For this reason, a new and expanded sampling effort was necessary to determine whether these results are apparent at a larger spatial extent.

The primary question addressed in this study is: Are models relating landscape characteristics to indices of nutrient loading dependent on aquatic ecosystem type? To address this question, consumers were collected from rivers and rivermouth ecosystems throughout the Great Lakes. The isotopic composition of these primary consumers was used as a time-integrated indicator of N loading [4]. Previous work has established a strong relationship between agricultural activities and the δ^15^N in tissues of aquatic consumers [4], [5], [8], [14]. We predict agricultural land cover and consumer tissue δ^15^N will have a positive linear relationship, as observed previously [8]. Some previous work has also suggested that low-flow aquatic habitats tend to be N sinks due to denitrification and sedimentation, which might also influence δ^15^N in consumer tissues [7], [8], [15]. We predict that land cover indicators of depositional or low-flow habitats such as wetlands and open surface waters (i.e., lake area) will be negatively related to consumer tissue δ^15^N. Finally, we predict that the magnitude of land cover effects on consumer tissue δ^15^N will vary with ecosystem type (river and rivermouth). Aquatic processes that remove or retain N are more effective in lower-flow environments [7], and by definition rivermouths are areas where lotic waters merge and become more lentic [10], [11]. Removal of agricultural N in rivermouths would lessen the strength of relationships between watershed agriculture and the N present in consumers (as observed previously [8]).

## Materials and Methods

### Ethics statement

No permits were required for the sample collection described herein. All of the sites sampled here are publicly accessible and no threatened or endangered species were collected as a part of this study.

### Study Sites

**Figure 1:**
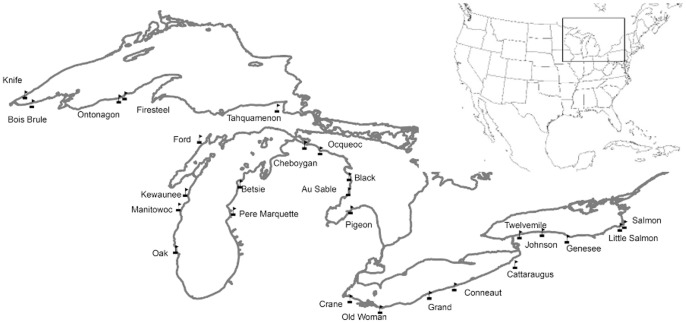
Map of sites sampled during this study.

Twenty-five tributary systems of the Laurentian Great Lakes were sampled during June-August of 2011 (Figure****1, Table****1). Two sites in each of these tributary systems were sampled: 1) At an upstream location outside the influence of seiche-driven lake water inputs (River; R) and 2) at the outlet where the rivermouth entered the adjacent lake (RM). R and RM sites were separated by an average of 7.7 river km (range 0.9–17.8; Table****1). Site selection was constrained by logistical issues: Sampling in Canada was not permitted and many rivers and rivermouths were inaccessible with our field equipment. We also excluded any site with obvious surface water inputs between R and RM sampling locations. Of the remaining sites, all sites with long-term discharge monitoring were sampled and additional sites were randomly selected to reach at least 5 sites per Great Lake. We also sampled Oak Creek (WI) opportunistically. Aerial photographs were used to confirm that there were minimal surface water inputs between our R and RM sites. At each site conductivity, pH and temperature were measured using a YSI probe once at the time of consumer sampling (pH calibrated daily; Model no. 600XLM).

**Table 1:** Study sites and characteristics.

Site	Date	Ag	WDep	RMDep	RM consumers	Distance from R to RM (river km)
Ford (MI)	8/22–8/23/11	4.2	47.4	0.4	DM	14.8
Kewaunee (WI)	6/21–6/23/11	78.1	6.2	1.4	DM	8.9
Manitowoc (WI)	8/29–8/30/11	69.3	15.0	0.1	DM	8.9
Pere Marquette (MI)	8/9/11	12.2	15.8	1.7	DM	16.2
Betsie (MI)	7/6–7/7/11	7.7	24.7	1.2	DM	10.1
Tahquamenon	6/16/2011				–	
Cheboygan	6/14/11	6.8	26.0	0.2	CF, DM	3.2
Ocqueoc	6/15/11	6.2	33.0	1.4	CF	0.9
Little Salmon (NY)	6/27/11	13.9	16.7	1.3	CF	4.8
Salmon (NY)	6/26–6/28/11	3.8	19.6	0.6	CF	7.7
Knife River (MN)	7/12–7/13/11				–	
Bois Brule (WI)	7/12–7/13/11				–	
Ontonagon (MI)	7/13–7/14/11	3.8	21.9	0.3	CF	5.0
Conneaut (OH)	7/20/11	32.5	5.9	0.9	DM	7.1
Grand (OH)	7/21/11	33.8	8.9	0.2	DM	13.6
Cataraugas (NY)	7/26–7/27/11	35.4	2.7	0.5	CF, DM	17.8
Genesee (NY)	6/28–6/29/11	45.9	4.6	0.0	DM	9.6
Twelvemile (NY)	7/27–7/29/11	66.5	5.3	5.3	DM	4.8
Johnson (NY)	7/28–7/29/11	59.4	9.2	0.9	DM	8.6
Pigeon (MI)	8/1/11	79.4	7.7	4.2	DM	5.0
Black (MI)	8/3/11				–	3.3
Au Sable (MI)	8/2–8/4/11	3.2	14.9	0.6	DM	3.0
Crane (OH)	8/10–8/11/11	72.1	11.3	11.1	DM	9.9
Old Woman (OH)	7/19/11	68.8	1.4	1.2	DM	5.6
Firesteel (MI)	8/24/11				–	
Oak (WI)	8/30–8/31/11	13.0	4.4	4.4	DM	1.2

Land cover (in percentage) is presented for the watershed upstream of the rivermouth (RM).

Ag  =  % of watershed with agricultural land cover; WDep  =  % of watershed covered by low-flow aquatic habitats (lakes +.

wetlands); RMDep  =  % of watershed covered by low-flow aquatic habitats below the R site; CF  =  caddisflies; DM  =  dreissenid mussels.

CF consumers were collected at all R sites. DM were collected at Cheboygan and Genesee R sites. Watershed land cover is not reported for sites where filter-feeding consumers could not be found.

### Consumers

At each location (R, RM), filter-feeding consumers were collected that would imply the isotopic composition of material entering the base of the food web. When possible, dreissenid mussels (either *Dreissena polymorpha* or *D. bugensis*) were collected, but dreissenids did not occur at many of the R sites, so Hydropsychidae caddisflies were also collected. All individuals were morphologically identified as *Dreissenna polymorpha*, although cryptic species variation cannot be ruled out [16]. No filter feeding basal consumers were available at a few locations, and these sites are excluded from the following analysis.

To evaluate the similarity of δ^15^N in caddisflies and dreissenids, samples of both taxa were collected whenever possible (5 sites). To strengthen this cross-taxa comparison, both caddisflies and dreissenids were collected from 7 nearshore lake sites (off shorelines and the outside walls of harbor walls). These nearshore lake sites were close to the RM sites for the Ford, Manitowoc, Cheboygan, Little Salmon, Cataragas, 12-Mile and Au Sable (sites and data in File S1).

Dreissenids and caddisflies were typically collected off of breakwalls and rocks within the outlet of the rivermouth, although some were found on woody debris. Consumers at R sites were collected from hard substrates in areas with flowing water. In smaller streams, these were collected from near the thalweg, but in larger streams where wading was not possible these individuals were taken from rocks and woody debris along the shoreline. All individuals were collected by hand. Target size for dreissenids was from ∼2–3****cm (to insure enough sample material), but size was not recorded and a few individuals outside of this range may have been collected. All consumers were taken from less than 1****m depth. A minimum of 3 dreissenids or 5 caddisflies were collected and grouped into a single sample. Consumers were kept chilled in coolers with ice until they could be processed (∼6****hours). Dreissenids had shells and byssal threads removed, and soft tissues were placed in cryovials prior to storage in liquid nitrogen. Caddisflies were placed whole in cryovials and then stored in liquid nitrogen.

### Stable Isotope Analysis

Consumers were stored in the field in liquid nitrogen, and then returned to the lab. In the lab, samples were stored in an −80°C freezer until they were lyophilized and shipped to the Colorado Plateau Stable Isotope Laboratory (http://www.mpcer.nau.edu/isotopelab/isotope.html) for analysis. Samples were analyzed using a CE Instruments NC2100 Elemental Analyzer interfaced to a Thermo-Electron Delta V gas-isotope ratio mass spectrometer. Analytical duplicates indicate small analytical error rates, with standard deviation <±0.1 ^0^/_00_ for δ^13^C and δ^15^N.

### Land cover

The watershed properties for the 23 rivermouths (where consumers were found) are based on watershed boundaries from the USGS Watershed Boundary Dataset (WBD) and user defined boundaries created from 10-meter DEMs from the USGS National Elevation Dataset (NED). The entire watershed basin properties (above the RM sampling location) were calculated based on the WBD and the properties above the R locations were based on those created from 10-meter NED using Pour Point in ArcGIS 10.0 [17]. After the watershed was created, summaries of land cover based on the 2006 National Land Cover Database were created [18]. From the full summaries, two categories were created: Agriculture and Depositional. The Agriculture category was the Cultivated Crops class plus the Pasture/Hay class. The Depositional category was the combination of Surface Water, Woody Wetlands and Emergent Wetlands classes.

The depositional areas specifically associated with the rivermouths below the R were estimated by subtracting the area of depositional habitat of the whole watershed at the RM site by the area of depositional habitat occurring above the R site. This is referred to as the RM depositional.

The area of the rivermouth itself was also estimated. The lake-ward boundary was across the outlet or harbor outlet for rivermouths with harbor walls that extend into the lake. For many tributary rivers, as the river approaches the lake, the river widens and backwater areas and islands become apparent in aerial photographs. The upstream boundary was placed at the most downstream point where the river still appeared to be in a constrained channel without these obvious backwater areas.

### Statistical analyses

All statistical analyses were conducted in R (version 2.11.1 [19]). Bayesian statistics were conducted using the BRugs package, which interfaces R to OpenBUGS [20]. Examples and descriptions of the code used for statistical analysis are provided in File S2. Mean values and 95% credible intervals of seston and consumer FAs at R, RM and L sites were made using the approach described in McCarthy, pp 66–67 [21] (see example code in File S2). Comparisons between R, RM and L sites in mean FA values were made by comparing the overlap of 95% credible intervals around the mean. In this approach, a ‘significant’ difference is inferred when 95% credible intervals do not overlap.

Although a single taxonomic group could be sampled at all R sites (the Hydropsychidae family), both RM sites had a mix of Hydropsychidae caddisflies and dreissenid mussels. To determine whether or not dreissenid and caddisfly consumers had similar tissue δ^15^N, a simple linear regression (the lm() function in R) was performed. A total of 13 pairs of caddisflies and dreissenids were used to evaluate this model. Since the model fit was strong (see Results), the regression model was used to convert dreissenid tissue δ^15^N from sites with only dreissenids into equivalent caddisfly tissue δ^15^N values. Other possible sources of variation in this model could not be evaluated with the available data (e.g., whether this relationship varies by ecosystem type).

The support for models relating land cover data and consumer δ^15^N was assessed using the deviance information criterion (DIC; [21]). The use of DIC is analogous to the use of the more common Akaike's information criterion (AIC; [22]). DIC differences (ΔDIC) are used to estimate the rank of the fit of the models to reality [21], with ΔDIC ≤2 indicating substantial support, ΔDIC from 4–7 indicating ‘considerably less’ support and ΔDIC >10 indicating essentially no support [21], [23]. Mean and 95% credible intervals were estimated for model parameters of models with ΔDIC <5.0. The Bayesian correlation coefficient (R^2^_B_) and its 95% credible interval were also estimated [24]. Credible intervals are similar to confidence intervals (see discussions in [21], [25], [26]). Together, estimates of ΔDIC, R^2^_B_ and model parameters indicate different aspects of statistical significance in these models (best model, the amount of variation being explained and the effect size, respectively). Example code used for this analysis is included in File S2. Several models were evaluated for significance. These linear models related variation of either agriculture, depositional areas or a combination of these two effects on consumer tissue δ^15^N. The agriculture data was included either untransformed or natural log-transformed (base *e*) agriculture (log*_e_*[% agriculture+1]). Logarithmic transformation was evaluated after data were plotted and a non-linear relationship seemed apparent. Depositional area data was untransformed.

The use of a Bayesian approach has several advantages on theoretical grounds that have been described elsewhere [21]. Pragmatically, this approach is ideal for this study as it allows the explicit incorporation of previously collected data into a given analysis. The data from Larson et****al. [8] was used to create the prior distributions for model parameters and precision (see File S2). To do this, models from Larson et****al. [8] were re-estimated using the Bayesian approach described here. To be sure the statistical method used here would not alter the conclusions of Larson et****al. [8], the previous data was completely re-analyzed using this statistical approach (File S3).

## Results

### Variation in δ^15^N among sites, habitat types and taxa

**Figure 2:**
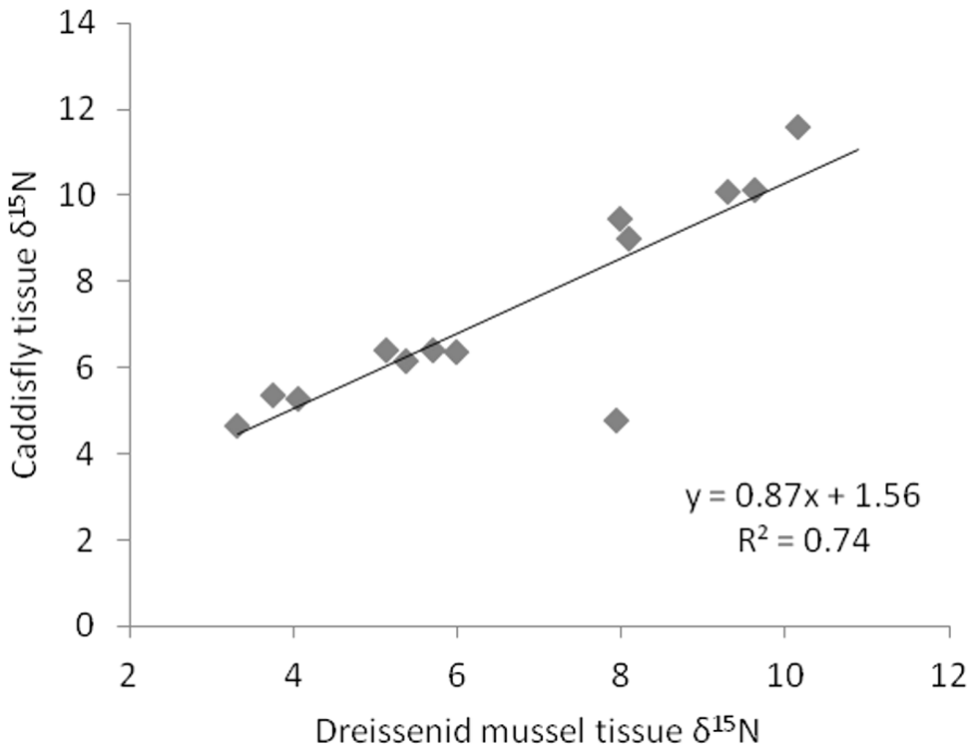
Relationship between caddisfly and dreissenid mussel tissue δ^15^N. Each point represents a location where both caddisflies and dreissenid mussels were collected.



There was considerable variation in the δ^15^N of the consumers sampled in this study (see File S1). Caddisfly tissue δ^15^N values ranged from 2.18 to 12.4 (R mean  = 7.93±2.64 [standard deviation], RM mean  = 6.87±2.12), while dreissenid mussel tissue δ^15^N ranged from 3.74 to 10.89 (R mean  = 6.53±3.28, RM mean  = 7.98±1.80). The δ^15^N of consumer tissues in rivers and rivermouths could not be directly compared because of taxonomic differences between these habitat types. Caddisflies were found at all R sites, but dreissenids were only found at 2 R sites (Table****1). Similarly, dreissenids were found at most RM sites, but at 5 sites only caddisflies could be found. Among-site variation in caddisfly and dreissenid tissue δ^15^N was strongly correlated, but caddisfly tissues had higher δ^15^N values (R^2^  = 0.74; Figure****2). The following regression model was used to convert dreissenid δ^15^N values from sites with only dreissenids into equivalent caddisfly δ^15^N values.

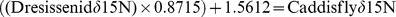



This conversion places caddisflies and dreissenids in a common framework in regards to tissue δ^15^N. For rivermouth consumers, the tissue δ^15^N in this common framework was used as the consumer tissue δ^15^N.

### Relationships between land cover and consumer tissue δ^15^N

**Figure 3:**
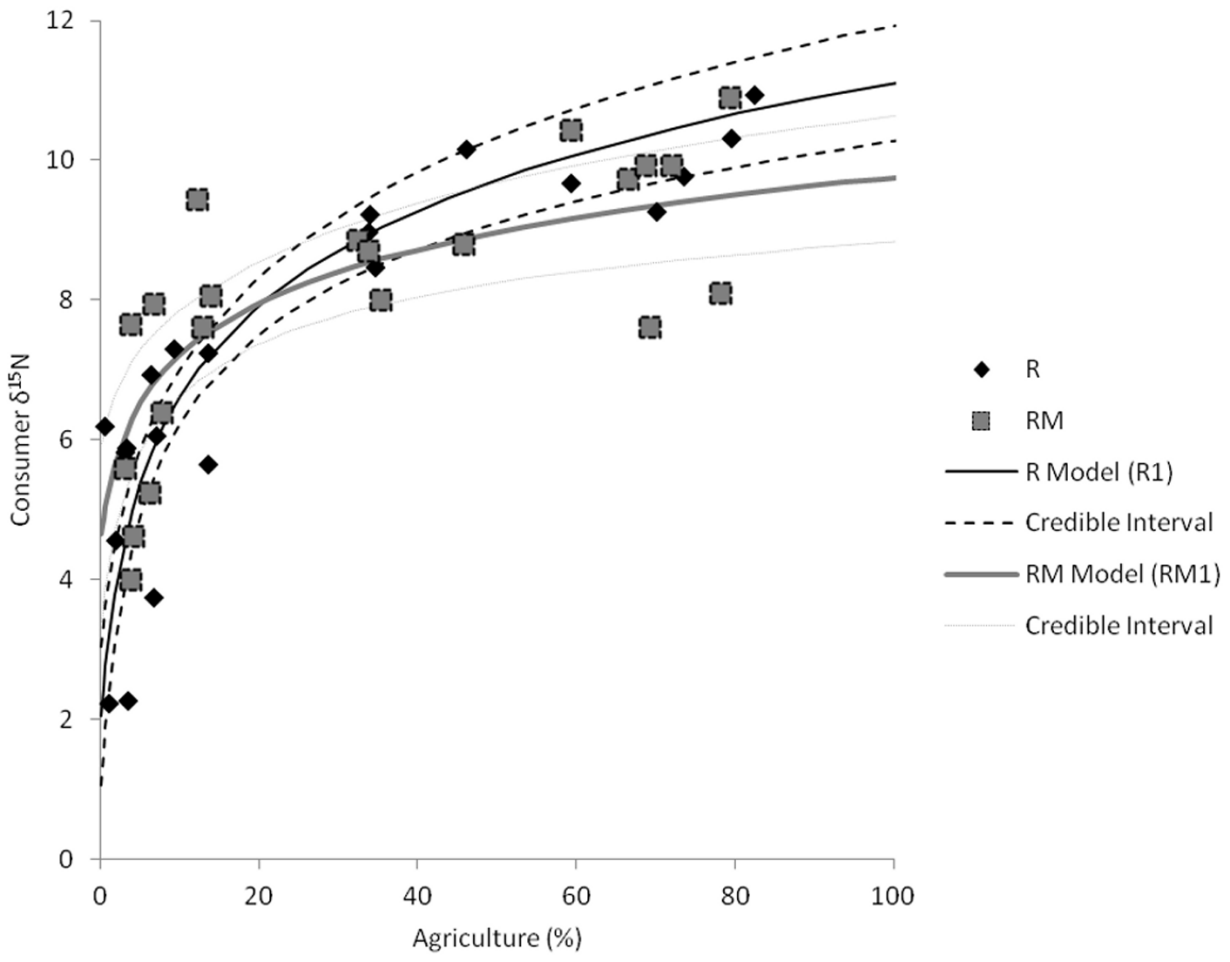
Modeled relationships between log*_e_*-transformed watershed agriculture and consumer tissue δ^15^N as estimated by the most strongly supported models (models R1 and RM1 from Table 2). Squares denote data from rivermouth (RM) consumers, diamonds are river (R) consumers. Solid lines denote the relationships between agriculture) and consumer δ^15^N for each of R and RM sites. The black line and black dashed lines are model and 95% credible intervals for the relationship derived at the R sites. For the purpose of this figure, the watershed depositional areas are held constant at 16.9% (the overall average for R sites). The grey dashed line and dotted lines are model and 95% credible intervals for the relationship derived at RM sites. Parameter values are listed in Table 2. 95% credible intervals in model estimates incorporate variation in both slope and intercept.

**Figure 4:**
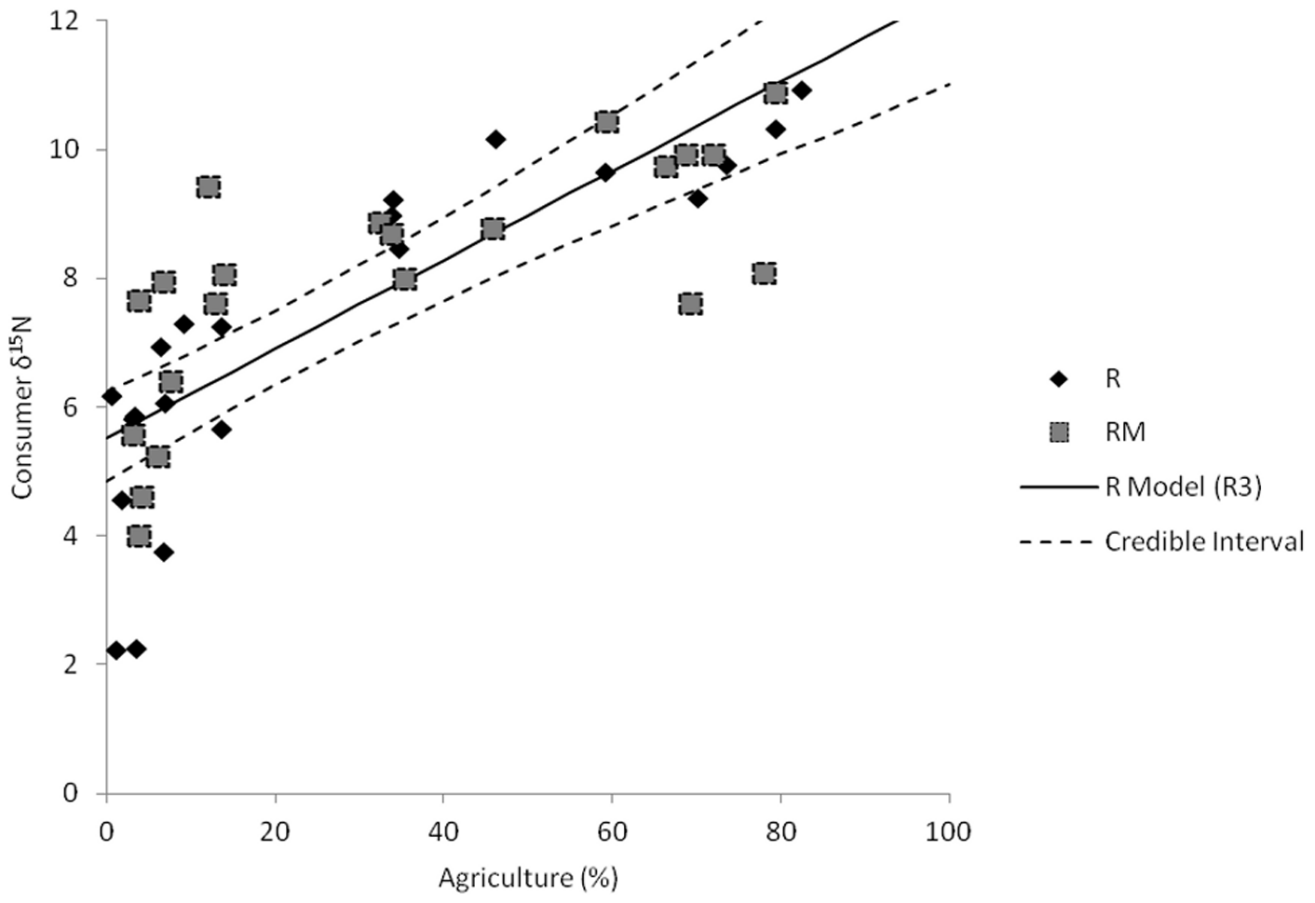
Relationship between watershed agriculture and consumer tissue δ^15^N. Squares are rivermouth (RM) consumers, diamonds are river (R) consumers. The lines represent the linear relationship between agriculture and consumer δ^15^N at the R sites (with 95% credible interval). The linear relationship for the RM sites was not statistically significant (parameters in Table 2).

At R sites, one model (R1) had substantial support (ΔDIC <2; Table 2) and a second model (R2) was also supported to a somewhat lesser degree (ΔDIC ∼2.9; Table 2). These models both suggested agriculture had an association with consumer tissue δ^15^N, and the nature of this relationship appeared to be logarithmic (Table 2, Figure****3). The proportion of depositional areas in the watershed is also included in the best model, but the addition of depositional areas only marginally increases the R^2^_B_ from 0.86 to 0.87 (Table 2). These two models have similar support from ΔDIC and R^2^_B_ indicates both explain about the same amount of variation. The magnitude of the agricultural effect in each is approximately the same (95% credible intervals for slope estimates overlap). There seems to be little substantial difference between the best two models at R sites. Equivalent models that did not use log*e-*transformed data had substantially less support (ΔDIC >5; Table 2, Figure 4), indicating the effect of agriculture is more likely to be non-linear.

**Table 2:** Results of model selection for relationship between watershed properties and δ^15^N in consumers and seston.

Location	Model No.	Model	ΔDIC	R^2^_B_
R	R1	1.1 _(−0.36 to 2.5)_ + WDep*0.041_(0.0095 to 0.072)_ + Ln(Ag)*2.0_(1.7 to 2.4)_	0	**0.87 (0.81 to 0.92)**
	R2	2.9_(2.0 to 3.7)_ +Ln(Ag)*1.6_(1.3 to 1.9)_	2.87	**0.86 (0.78 to 0.91)**
	R3	5.5_(4.8 to 6.2)_+Ag*0.069_(0.052 to 0.086)_	5.02	**0.67 (0.44 to 0.81)**
	R4	WDep + Ag	6.01	–
	R5	WDep	68.4	–
				
RM	RM1	4.5_(3.2 to 5.8)_ + Ln(Ag)*1.4_(0.73 to 1.54)_	0	**0.47 (0.10 to 0.69)**
	RM2	5.4_(2.5 to 8.2)_ + WDep*−0.043_(−0.11 to 0.023)_ + Ln(Ag)*1.0_(0.38 to 1.7)_	0.1	**0.47 (0.09 to 0.70)**
	RM3	8.1_(6.6 to 9.6)_ + WDep *−**0.07_(_**_−**0.13 to** −**0.014)**_ + Ag *0.027_(0.0035 to 0.051)_	2.39	0.42 (−0.01 to 0.66)
	RM3	6.5_(5.8 to 7.3)_ + Ag*0.042_(0.02 to 0.06)_	4.90	0.34 (−0.11 to 0.61)
	RM4	WDep	5.81	–
	RM5	RMDep + Ln(Ag)	8.71	–
	RM6	RMDep+Ag	14.99	–
	RM7	RMDep	25.89	–

Estimates of intercepts, coefficients and R^2^_B_ values (with 95% credible intervals) are shown only for models with a ΔDIC of less than 5. R  =  river sites; RM  =  rivermouth sites; Ag  =  percentage of watershed that is agriculture; WDep  =  percentage of watershed that is depositional habitats (lakes plus wetlands); RMDep  =  depositional habitats below the R site as a percentage of the entire watershed; Ln(Ag)  =  the natural log of Ag plus 1.

At RM sites, the model selection procedure yielded largely similar results (Table 2). The two strongly supported models both included the logarithmic association with agriculture and barely differed in DIC or R^2^_B_ (Table 2, Figure 3). The difference between these models was caused by the inclusion of whole watershed depositional areas. However, the slope estimate for depositional areas was negative (opposite expectations [4] and estimate in R1) and that parameter estimate was not statistically different from zero (95% credible interval −0.11 to 0.023; Table 2). Untransformed agricultural data in the models also resulted in models with some support (ΔDIC <5; Table 2), although R^2^_B_ were lower. Using just the depositional areas associated with the rivermouth instead of using the depositional areas of the entire watershed created weaker models (models with RMDep instead of WDep were not strongly supported).

Comparing the models of association between agriculture and consumer tissue δ^15^N at R and RM sites reveals some differences (Table 2). The most strongly supported model using R site data has a log*_e_*-transformed agriculture parameter coefficient (R1  = 2.0_[1.7–2.4]_; 95% credible intervals in brackets) that is significantly higher than the either of the strongest models created using RM data (RM1 = 1.4_[0.73–1.54]_ and RM2  = 1.0_[0.38–1.7]_; Table 2). However, these models still have broad overlap in credible intervals (incorporating variation in both slope and intercept; Figure 3). Also differing between the best R and RM models is in the amount of variation being explained. Models derived from R data have a significantly higher R^2^_B_ (R^2^_B_  = 0.87_[0.81–0.92]_) than models derived using RM data (R^2^_B_  = 0.47_[0.10–0.69]_) and a narrower credible interval on the agricultural parameter coefficient (R1  = 0.58; RM1  = 0.81; RM2  = 1.32). For these reasons, the RM data suggest a weaker relationship between agriculture and consumer tissue δ^15^N than the R data.

### Relationships from Larson et al. [8]

In Larson et****al. [8] only untransformed agriculture data were used to relate agriculture and depositional areas to consumer δ^15^N. Those models were not among the most strongly supported in the current analysis (Table 2). Including log*_e_*-transformed agriculture data in the analysis of the earlier dataset would not qualitatively change the inferences of Larson et al. [8] because the earlier data included too few sites to adequately characterize a logarithmic relationship (see results of such a re-analysis in File S3).

The other relationship identified in Larson et al [8] was one between rivermouth depositional areas (RMDep) and consumer tissue δ^15^N. Inclusion of rivermouth depositional areas did not improve model fit in the current dataset (even though that earlier data was explicitly incorporated into this analysis through the prior distributions). However, the relationship in Larson et al. [8] was largely driven by 2 sites. With this expanded dataset, that relationship is no longer among most supported models, even if only the linear models are considered.

## Discussion

### Agricultural land cover and consumer tissue δ^15^N

Agriculture and consumer tissue δ^15^N were strongly, positively related in this study, as has been observed in a variety of locations previously [4], [14], [27], [28]. This observation is consistent with the understanding that agricultural and urban sources of labile N either have a high δ^15^N or become more enriched by processing in aquatic ecosystems [4], [29]. The strong relationship between agriculture and the tissue δ^15^N of primary consumers is well-established on both empirical and theoretical grounds.

Somewhat unexpectedly, the nature of the relationship between agriculture and consumer tissue δ^15^N that was most strongly supported was not one that we initially considered. Indeed, the decision to look for a logarithmic model form (i.e., using log-transformed data) was driven by a visual inspection of the data rather than an *a priori* hypothesis. However, consumer tissue δ^15^N reflects agricultural land cover because agricultural N has a distinct δ^15^N composition [4], [29]. As agricultural N becomes an increasing component of the total N consumed, the consumer tissue δ^15^N more closely reflects that agricultural N isotopic composition. The relationship between agricultural land cover and N loading from agriculture is presumably linear, but this does not necessarily mean that biotic accumulation of this N is similarly linear [14]. If, for example, phosphorus (P) is limiting, then once the biotic demand from N is met, additional N inputs might not lead to further alterations in the isotopic N composition in biota. Similarly, if agricultural N is more labile than other N sources, then all biotic N demand may be met by agricultural N even if other N sources are available. For these reasons, a logarithmic model that approximates a saturation curve over the gradient of possible agricultural land cover values is mechanistically reasonable, although we are unaware of previous studies that have seen such a relationship.

### Depositional habitats and consumer tissue δ^15^N

Depositional habitats (wetlands and other low-flow areas) are locations where denitrification rates can be high and for this reason depositional habitats are thought to influence the overall movement of N across landscapes [7], [15]. Denitrification effectively removes N from aquatic systems by producing N_2_ (which is inert) and increases the δ^15^N of remaining N. Denitrification is such a widespread process that even fertilizer inputs (with an initial δ^15^N of ∼0) appear to have a positive association with consumer tissue δ^15^N [4]. However, other than our previous work [8], there do not appear to have been studies that explicitly investigated the relationship between watershed depositional areas (e.g., wetlands) and consumer tissue δ^15^N. The positive effect of depositional habitats observed here in river sites is conceptually consistent with the literature [15], [29], but this effect differs from that observed at rivermouth sites and in our previous work [8]. Other studies have used aggregated land cover variables (e.g., [5], [27]) that typically include wetlands on the opposite end of the spectrum from agriculture, implying that wetlands cause lower consumer tissue δ^15^N by not being a source of labile N. Obviously, for denitrification to have a significant effect on consumer tissue δ^15^N requires there to be N available to be denitrified. For this reason, landscape context may be an important consideration when assessing the impact of depositional habitats on δ^15^N in aquatic systems. Possibly, wetlands with “upstream” sources of N from agriculture will have a greater effect on food web δ^15^N than wetlands that do not have significant labile N inputs. This study lacks any measure of that landscape context, potentially explaining both the difference in results between this and other studies [8], as well as the inconsistency between results from the river and rivermouth consumer data.

### Are models relating landscape characteristics to indices of nutrient loading dependent on aquatic ecosystem type?

In contrast to our expectations [8], models relating land cover to consumer tissue δ^15^N were strongly supported at both river and rivermouth sites. Both the direction and overall shape of these models were very similar in both ecosystem types. Further, river and rivermouth consumers sampled did not appear to differ significantly in average tissue δ^15^N (after correction for taxonomic differences). At some level, this contradicts the results of our earlier work and suggests the answer to the primary question of this study is ‘no.’ More importantly, this result suggests that land cover does have a substantial influence over the δ^15^N in rivermouth food webs, and that this influence is mechanistically similar to the influence land cover has over river food webs.

However, the details of these models vary considerably between these ecosystem types. This is most easily seen by the amount of variation explained by the models, which is considerably lower at rivermouth sites, indicating land cover models are less predictive of actual consumer tissue δ^15^N in rivermouth ecosystems. The magnitude of the agricultural land cover coefficient is lower in rivermouths and both magnitude and direction of the depositional habitat coefficient differs between river and rivermouth models. The variation in predictive capability is consistent with other studies: Peterson et****al. [5] saw the R^2^ of simple linear models relating agriculture to benthic consumer tissue δ^15^N was greater in coastal wetlands (0.82) than in adjacent nearshore waters (0.47). Whether the diminished strength of this relationship at the rivermouth is because of processing within these mixing zones, simple mixing with nearshore zone waters or because of some other factor is unclear.

The evidence presented here does suggest ecosystem-specific differences in the relationship between agriculture and consumer tissue δ^15^N. However, important caveats from this analysis should be kept in mind. For example, there is no way to effectively evaluate the possible effects of taxonomic variation as a cause of the reduced relationship at the rivermouth sites. For river sites, all consumers came from a single family (Hydropsychidae caddisflies), whereas rivermouth consumers were a mix of caddisflies and dreissenid mussels. Although tissue δ^15^N varied similarly among sites for caddisflies and dreissenid mussels, species-specific differences may have had an effect on the ability of the best models to relate agriculture to consumer tissue δ^15^N.

## Conclusions

Consumers can be considered time-integrated samplers of their available resources, making them excellent indicators of the abundance of distinct resources. In this context, previous work has suggested using primary consumers as indicators of agricultural N, which has a distinct isotopic signature (e.g., [4]). This concept makes the most sense conceptually in stream and river systems, where water residence times are low, and aquatic processes have little opportunity to alter nutrient loads and isotopic signatures. As rivers approach rivermouths, waters slow, suspended loads are deposited and water residence times increase [11], all factors that can alter N dynamics and isotopic signature [4], [7]. Consistent with this reasoning, we found strong relationships between consumer N and agriculture in upstream watersheds in stream and river sites, but somewhat weaker relationships in those same tributaries at the outlet to the nearshore zone of large lakes. This result implicitly suggests that significant N processing (or sources) occur in rivermouths that do not occur in rivers.

The identity of these rivermouth-specific processes (or sources) is unclear. Earlier work suggested depositional habitats associated with the rivermouth were driving this additional variation in N dynamics in rivermouths [8], but this study offers no support for that conclusion. Rivermouths do receive regular inputs of nearshore zone water from seiche activities [30] and these inputs might transport significant quantities of N to the rivermouth. Further, these seiche events cause river water to slow, potentially increasing the likelihood of denitrification [7], [31]. Few studies have documented the dynamics of water mixing in rivermouths, but those that have demonstrate that mixing regimes vary dramatically in response to season, storm events and differences in water density [11], [30]–[33]. This mixed hydrology lacks a riverine analog and seems capable of significantly altering N dynamics (thus influencing δ^15^N composition in the food web). Determining whether among-system variation in the frequency or extent of seiche-driven lake influences alter N dynamics in rivermouths should be a focus of future research efforts.

Many estimates of nutrient loading from rivers to coastal zones occur upstream of rivermouths (and estuaries; [9]). This study suggests processes or sources within rivermouths are altering N dynamics. This raises the possibility that estimates of nutrient loading to the nearshore zone are inaccurate. Determining the likelihood and magnitude of systematic inaccuracies in these loading estimates will require more detailed hydrologic and biogeochemical modeling of the rivermouth itself, or validated indices of these processes.

## Supporting Information

File S1 **Raw and transformed data used in this analysis.** Six spreadsheets are included here, with the spreadsheet labeled “MetaData” explaining what the other spreadsheets contain.
(XLSX)

File S2 **Statistical appendix.** This supplemental file includes a description of the model selection procedure, a description of the procedure used to generate Figures****4 and 5 and a description of the process used to generate the mean and 95% credible intervals presented in Figure****3.
(DOCX)

File S3 **Table showing results of the present data analysis performed on data previously published in Larson et al.** [**8**] **.**
(DOCX)
