# Supplementary material for: Rivermouth Alteration of Agricultural Impacts on Consumer Tissue δ15N
Source: PLoS One. 2013 Jul 31;8(7):e69313. doi: 10.1371/journal.pone.0069313 (PMC3729966; doi:10.1371/journal.pone.0069313)
Supplement: File S2 — Statistical appendix. This supplemental file includes a description of the model selection procedure, a description of the procedure used to generate Figures4 and 5 and a description of the process used to generate the mean and 95% credible intervals presented in Figure3. (DOCX) [file pone.0069313.s002.docx]

**S2. Statistical Appendix.**

Several distinct statistical procedures are described here. All statistical analyses were conducted in R (version 2.11.1; [1]). Bayesian statistics were conducted using the BRugs package, which interfaces R to OpenBUGS [2]. All approaches follow methods described by McCarthy [3]. This appendix is presented in three parts: A description of the model selection procedure (A.), a description of the procedure used to generate Figures 4 and 5 (B.) and a description of the process used to generate the mean and 95% credible intervals presented in Figure 3 (C.).

*A. Model selection*

In this model selection procedure, a comma-separated values (.csv) file of the data (which corresponds to a spreadsheet in the Data Appendix) is attached so that R can 'see' the individual columns as variables. In the example code below, AllAg (all forms of agricultural land cover) is natural-log converted prior to analysis. As a result, in this example the DIC and parameter estimates are made for a model logarithmically relating agricultural land cover to the consumer tissue δ^15^N (FF15N). The general format for the regression equation was:

Consumer tissue δ^15^N = WatershedProperty(β) + Intercept

library("BRugs") ## This loads the BRugs package

Year1RiverFF <- read.csv("C:/Users/jhlarson/Desktop/USGS Science/Stable Isotopes 2011/Analysis/R Model Selection/Year1RiverFF.csv") ## Loading the dataset

attach(Year1RiverFF) ## attaching an imported dataset

LogAllAg <- log(AllAg+1)

## Regression Model - This creates a function that BRugs can use in OpenBUGS

regressionmodel <- function(){

a~dnorm(0,1.0E-6) ## Non-informative prior y-intercept

b~dnorm(0,1.0E-6) ## Non-informative prior slope

prec~dgamma(0.001,0.001) ## Non-informative model precision

sy2 <- pow(sd(y[]),2)

R2B <- 1 - 1/(prec*sy2) ## Bayesian R2

for (i in 1:11) ## 1:N, where N is the number of observations

{

mean [i] <- a+b*x[i]

y[i] ~dnorm(mean[i],prec)

}

}

regressionmodelfile <- file.path(tempdir(),"regressionmodel.txt")

model <- writeModel(regressionmodel,regressionmodelfile)

inits <- "C:\\Users\\jhlarson\\Desktop\\USGS Science\\OpenBugs Code\\Regression\\Initials.txt"

## location of the initials file: a=0, b=0, prec=100

## Test for AllAg.

x <- LogAllAg ## for convenience variable is converted to x to match model

y <- FF15N ## for convenience variable is converted to y to match model

bdata <-bugsData(c("y","x"),,digits=5) ## this places the data into a form OpenBUGS can read

modelCheck(regressionmodelfile) ## Tells OpenBUGS to check the model

modelData(bdata) ## Tells OpenBUGS to load the data

modelCompile(numChains=1) ## Compiles the model with number of chains

modelInits(inits,) ## Loads the initials

modelUpdate(50000) ## Updates the model 50000 times as a burn-in

samplesSet(c("b","R2B","a","prec"))## Tells OpenBUGS to keep data on these variables

dicSet() ## Tells OpenBUGS to keep data on DIC

modelUpdate(50000) ## Updates model 50000 times to collect data

YR1R15NLogAllAg <- samplesStats("*") ## Store the variable estimates in a designated file

YR1R15NLogAllAgDIC <- dicStats() ## Store the DIC estimates in a designated file

This basic approach was repeated for every model tested. The model above could be used for any 1-parameter model. However, for 2-parameter models, this procedure was slightly modified by the addition of a new model:

## Two-parameter Regression Model

regressionmodel <- function(){

a~dnorm(0,1.0E-6)

b1~dnorm(0,1.0E-6)

b2~dnorm(0,1.0E-6)

prec~dgamma(0.001,0.001)

sy2 <- pow(sd(y[]),2)

R2B <- 1 - 1/(prec*sy2)

for (i in 1:11)

{

mean [i] <- a+b1*x1[i]+b2*x2[i]

y[i] ~dnorm(mean[i],prec)

}

}

regressionmodelfile <- file.path(tempdir(),"regressionmodel.txt")

model <- writeModel(regressionmodel,regressionmodelfile)

inits <- "C:\\Users\\jhlarson\\Desktop\\USGS Science\\OpenBugs Code\\Regression\\Initials2variables.txt"

x1 <- Wdep

x2 <- LogAllAg

y <- FF15N

bdata <-bugsData(c("y","x1","x2"),,digits=5)

In this example, initials were set at a=0, b1=0, b2=0 and prec=100. Other aspects of the procedure were identical, except that parameters a,b1,b2, R2B and prec were monitored.

The above models have non-informative prior distributions, and these models were used on the data from Larson et al. [4] to generate informative prior distributions that could be used in the analysis of the new data. This re-analysis of the earlier data is summarized in Appendix Table 1. These distributions were used as informative priors in the analysis of the new data. In the example below, data from Appendix Table 1 is used to create prior distributions for model parameters and model precision.

attach(RiverFF)

LogAllAg <- log(AllAg+1)

## Regression Model

regressionmodel <- function(){

a~dnorm(5.213,0.5259)

b~dnorm(1.497,5.4641)

prec~dgamma(13.164,4.5087)

sy2 <- pow(sd(y[]),2)

R2B <- 1 - 1/(prec*sy2)

for (i in 1:22)

{

mean [i] <- a+b*x[i]

y[i] ~dnorm(mean[i],prec)

}

}

regressionmodelfile <- file.path(tempdir(),"regressionmodel.txt")

model <- writeModel(regressionmodel,regressionmodelfile)

inits <- "C:\\Users\\jhlarson\\Desktop\\USGS Science\\OpenBugs Code\\Regression\\Initials.txt"

## Test for AllAg.

x <- LogAllAg

y <- FF15N

bdata <-bugsData(c("y","x"),,digits=5)

modelCheck(regressionmodelfile)

modelData(bdata)

modelCompile(numChains=1)

modelInits(inits,)

modelUpdate(50000)

samplesSet(c("a","b","R2B","prec"))

dicSet() ## Tells OpenBUGS to keep data on DIC

modelUpdate(50000)

R15NLogAllAg <- samplesStats("*")

R15NLogAllAgDIC <- dicStats() ## Store the DIC estimates in a designated file

*B. Visualizing the model and 95% credible intervals*

Creating a visual representation of the model plus 95% credible intervals can be done by creating predictions from the model across the parameter space and displaying those predictions in a graphic. Although not necessarily the best mechanism to do this, we made these estimates in R using the BRugs, then exported the resulting estimates to Excel to build a figure. In this example, we calculated predictions for the range of possible values following a procedure suggested by McCarthy [3].

RiverFF <- read.csv("C:/Users/jhlarson/Desktop/USGS Science/Stable Isotopes 2011/Analysis/R Model Selection/RiverFF.csv")

attach(RiverFF)

LogAllAg <- log(AllAg+1)

## Regression Model

regressionmodel <- function(){

a~dnorm(5.213,0.5259)

b~dnorm(1.497,5.4641)

prec~dgamma(13.164,4.5087)

prediction0.15<-a+b*0.15

## This generates the prediction for a particular value

prediction0.25<-a+b*0.25

prediction0.35<-a+b*0.35

prediction0.5<-a+b*0.5

prediction1<-a+b*1

prediction1.6<-a+b*1.6

prediction1.8<-a+b*1.8

prediction2<-a+b*2

prediction2.2<-a+b*2.2

prediction2.4<-a+b*2.4

prediction2.6<-a+b*2.6

prediction3<-a+b*3

prediction3.1<-a+b*3.1

prediction3.2<-a+b*3.2

prediction3.3<-a+b*3.3

prediction3.4<-a+b*3.4

prediction3.5<-a+b*3.5

prediction3.6<-a+b*3.6

prediction3.7<-a+b*3.7

prediction3.8<-a+b*3.8

prediction3.9<-a+b*3.9

prediction4<-a+b*4

prediction4.1<-a+b*4.1

prediction4.2<-a+b*4.2

prediction4.3<-a+b*4.3

prediction4.4<-a+b*4.4

prediction4.45<-a+b*4.45

prediction4.5<-a+b*4.5

prediction4.55<-a+b*4.55

prediction4.6<-a+b*4.6

prediction4.65<-a+b*4.65

prediction5<-a+b*5

sy2 <- pow(sd(y[]),2)

for (i in 1:22)

{

mean [i] <- a+b*x[i]

y[i] ~dnorm(mean[i],prec)

}

}

regressionmodelfile <- file.path(tempdir(),"regressionmodel.txt")

model <- writeModel(regressionmodel,regressionmodelfile)

inits <- "C:\\Users\\jhlarson\\Desktop\\USGS Science\\OpenBugs Code\\Regression\\Initials.txt"

## Test for AllAg.

x <- LogAllAg

y <- FF15N

bdata <-bugsData(c("y","x"),,digits=5)

modelCheck(regressionmodelfile)

modelData(bdata)

modelCompile(numChains=1)

modelInits(inits,)

modelUpdate(50000)

samplesSet(c("a","b","prediction0.15","prediction0.25","prediction0.35","prediction0.5","prediction1","prediction1.6","prediction1.8","prediction2","prediction2.2","prediction2.4","prediction2.6","prediction3","prediction3.1","prediction3.2","prediction3.3","prediction3.4","prediction3.5","prediction3.6","prediction3.7","prediction3.8","prediction3.9","prediction4","prediction4.1","prediction4.2","prediction4.3","prediction4.4","prediction4.45","prediction4.5","prediction4.55","prediction4.6","prediction4.65","prediction5"))

modelUpdate(50000)

LogAgPredictions <- samplesStats("*")

*C. Descriptive statistics*

Estimating a mean using a Bayesian approach includes estimation of 95% credible intervals [3]. This makes for a simple test of statistically significant differences: If intervals overlap, then the means are not different. The following code was used to estimate mean and 95% credible intervals for consumer tissue δ^15^N.

## First the data file is loaded.

RiverFF <- read.csv("C:/Users/jhlarson/Desktop/USGS Science/Stable Isotopes 2011/Analysis/R Model Selection/RiverFF.csv")

attach(RiverFF) ## This allows R to read columns as variables

## This defines the function for OpenBUGS

regressionmodel <- function(){ ## the naming of this function is arbitrary

for (i in 1:22){

x[i] ~ dnorm (mu[1], tau[1])

}

mu[1] ~ dnorm (0, 0.0001) ## non-informative prior distributions were used

tau[1] ~ dgamma (0.001, 0.001)

}

regressionmodelfile <- file.path(tempdir(),"regressionmodel.txt")

model <- writeModel(regressionmodel,regressionmodelfile)

inits <- "C:\\Users\\jhlarson\\Desktop\\USGS Science\\OpenBugs Code\\1meansinitials.txt" ## this is the location of the initials file

x <- FF15N ## Transforming the variable to the same term used in the function

bdata <-bugsData(c("x"),,digits=5) ## This prepares the data in the format OpenBUGS uses

modelCheck(regressionmodelfile) ## These are the same as described above.

modelData(bdata)

modelCompile(numChains=1)

modelInits(inits,)

modelUpdate(50000)

samplesSet(c("mu","tau"))

modelUpdate(50000)

MeanFF15N<- samplesStats("*")

## The same process is repeated for the RM sites below

RMFF <- read.csv("C:/Users/jhlarson/Desktop/USGS Science/Stable Isotopes 2011/Analysis/RM Model Selection/RMFF.csv")

attach(RMFF)

## Regression Model

regressionmodel <- function(){

for (i in 1:21){

x[i] ~ dnorm (mu[1], tau[1])

}

mu[1] ~ dnorm (0, 0.0001)

tau[1] ~ dgamma (0.001, 0.001)

}

regressionmodelfile <- file.path(tempdir(),"regressionmodel.txt")

model <- writeModel(regressionmodel,regressionmodelfile)

inits <- "C:\\Users\\jhlarson\\Desktop\\USGS Science\\OpenBugs Code\\1meansinitials.txt"

x <- FF15N

bdata <-bugsData(c("x"),,digits=5)

modelCheck(regressionmodelfile)

modelData(bdata)

modelCompile(numChains=1)

modelInits(inits,)

modelUpdate(50000)

samplesSet(c("mu","tau"))

modelUpdate(50000)

RMMeanFF15N<- samplesStats("*")

**References**

1. R Development Core Team (2010) R: A language and environment for statistical computing.

2. Openbugs T, Best N, Lunn D (2007) The BRugs Package.

3. McCarthy M (2007) Bayesian methods for ecology. New York, New York, USA: Cambridge University Press. p.

4. Larson JH, Richardson WB, Vallazza JM, Nelson JC (2012) An exploratory investigation of the landscape-lake interface: Land cover controls over consumer N and C isotopic composition in Lake Michigan rivermouths. Journal of Great Lakes Research 38: 610–619.
