# Supplementary material for: Rivermouth Alteration of Agricultural Impacts on Consumer Tissue δ15N
Source: PLoS One. 2013 Jul 31;8(7):e69313. doi: 10.1371/journal.pone.0069313 (PMC3729966; doi:10.1371/journal.pone.0069313)
Supplement: File S3 — Table showing results of the present data analysis performed on data previously published in Larson et al. [8] . (DOCX) [file pone.0069313.s003.docx]

**S3. Models created using data from Larson et al. [8].**

| Location | Model | Precision | 𝜟DIC | R^2^_B_ |
| --- | --- | --- | --- | --- |
| R | *a* (6.963,1.8999) + Ag * β(0.07738,3777.7) | (8.5475,4.5079) | 0 | 0.66 (0.12 to 0.88) |
|  | *a* (7.837,0.2446) + Ag * β(0.07895,1252.1) + WDep * β(0.004251,323.95) | (8.4421,3.956) | 2.74 | 0.60 (-0.11 to 0.86) |
|  | *a* (5.213,0.5259) + Ln(Ag) * β(1.497,5.4641) | (13.164,4.5087) | 4.74 | 0.48 (-0.37 to 0.81) |
|  | *a* (3.407,0.0426) + Ln (Ag) * β(1.856,0.9408) + WDep * β(0.03795,109.53) | (12.674,3.9542) | 7.24 | 0.40 (-0.71 to 0.80) |
|  | *a* (11.88,0.6697) + WDep * β(-0.1148,414.8) | (20.227,4.5106) | 9.46 | 0.20 (-1.08 to 0.70) |
|  |  |  |  |  |
| RM | *a* (8.638,6.319321) + RMDep * β(-0.4705,102.1335) | (4.0520,4.5098) | 0 | 0.66 (0.12 to 0.88) |
|  | *a* (8.874,1.5022) + Ag * β(-0.005257,4103.8757) + RMDep * β(-0.496,58.7190) | (3.9409,3.9842) | 2.45 | 0.61 (-0.07 to 0.87) |
|  | *a* (9.199,0.4277) + Ln(Ag) * β(-0.1543,6.1512) + RMDep * β(-0.5142,40.6213) | (3.9092,3.9640) | 2.46 | 0.61 (-0.09 to 0.87) |
|  | *a* (5.153,0.5312) + Ln(Ag) * β(0.805,5.5704) | (11.3522,4.5114) | 11.32 | 0.05 (-1.5 to 0.65) |
|  | *a* (8.800,1.1468) + WDep * β(-0.06186,737.6196) | (12.1970,4.5092) | 12.12 | -0.02 (-1.6 to 0.63) |
|  | *a* (6.579,1.1419) + Ag * β(0.02909,2135.43) | (13.6424,4.5061) | 13.36 | -0.14 (-2.0 to 0.58) |
|  | *a* (5.501,0.0376) + Ln (Ag) * β(0.7317,0.7986) + WDep * β(-0.006275,113.68) | (11.2325,3.9617) | 14.09 | -0.12 (-2.2 to 0.62) |
|  | *a* (8.405,0.1765) + Ag * β(0.006518,817.72) + WDep * β(-0.05296,251.8727) | (11.9829,3.9567) | 14.81 | -0.19 (-2.3 to 0.59) |
| Models are shown with parameter estimates. For parameters with normal distributions (parameter coefficients and the y-intercept), mean and precision are reported. For model precision ("prec", modeled as a gamma distribution), *r* and *nu* are estimated. These are defined in McCarthy [3]. Ag = % of watershed with agricultural land cover; WDep = % of watershed covered by low-flow aquatic habitats (lakes + wetlands); RMDep = % of watershed covered by low-flow aquatic habitats below the R site; Ln(Ag) = Natural-log of the Ag data plus one; Ln(Ag+1). | | | | |
